# Impacts of medicinal cannabis on an early psychosis service

**DOI:** 10.1177/10398562231222818

**Published:** 2023-12-21

**Authors:** Katie Lupke, Amy Gerard, Brendan Murdoch, Nagaraj Gundarpi, Stephen Parker

**Affiliations:** 157827 Herston, QLD; 157827 Herston, QLD; 157827 Herston, QLD; 157827 Herston, QLD; 157827 Herston, QLD

Dear Editor,

The recent dramatic rise in the prescription of medicinal cannabis in Australia^1^ is not supported by growing evidence of its efficacy and effectiveness. The TGA guidance states, ‘medicinal cannabis products containing THC are generally **not appropriate** for patients who have a previous psychotic or concurrent active mood or anxiety disorder’.^2^ However, despite the limited evidence to support its use in the management of mental disorders,^3^ anxiety is one of the most frequent indications for prescription.^1^ Furthermore, young people are more likely to be prescribed high-dose ∆9-tetrahydrocannabinol (THC) products than formulations where cannabidiol (CBD) is prioritised. These trends are concerning given the established risks of youth cannabis use on mental health outcomes and neurocognition.^4^

At our Early Psychosis service, we have noted increasing referrals where medicinal cannabis has been contemporaneous with the onset of psychosis. Between 11/2022 and 07/2023, six of 67 patients referred had been using high concentrate prescribed THC in the preceding 3 months (own prescription (*n* = 5), family member prescription (*n* = 1)). Two of these consumers continued to obtain prescriptions *after* the onset of psychosis. Additionally, four consumers received a de novo prescription for high concentrate THC *after* psychosis onset. Prescriptions (most commonly THC (18%)/CBD (1%)) were mainly obtained through online services (66.7%); the indication was anxiety in all cases. Our opinion is that these prescriptions complicated the treatment and recovery from the first episode of psychosis.

We regularly have conversations with consumers and their families about the increasing public perception of medicinal cannabis as a harmless panacea being contrary to our clinical experience working at an early psychosis service. There is a concerning lack of regulation allowing consumers to source medicinal cannabis, often from interstate online prescribers, without comprehensive assessment.

If Australia continues to medicalise cannabis products, urgent efforts are needed to ensure evidence-based prescribing for mental disorders that achieves therapeutic benefit rather than iatrogenic harm. As emphasised in the RANZCP Clinical Memorandum – Therapeutic use of medicinal cannabis products (2021),^5^ this will require tightening regulatory processes and further research. There is a growing need for enhanced public awareness about the risks and benefits of medicinal cannabis for young people as well as people experiencing mental illness in general.

This project was assessed to be exempt from the requirement for ethical approval by the relevant Human Research Ethics Committee.
